# Fertilization of Ascidians: Gamete Interaction, Self/Nonself Recognition and Sperm Penetration of Egg Coat

**DOI:** 10.3389/fcell.2021.827214

**Published:** 2022-01-21

**Authors:** Takako Saito, Hitoshi Sawada

**Affiliations:** ^1^ Faculty of Agriculture Department of Applied Life Sciences, Shizuoka University, Shizuoka, Japan; ^2^ Depatment of Food and Nutritional Environment, College of Human Life and Environment, Kinjo Gakuin University, Nagoya, Japan; ^3^ Graduate School of Science, Nagoya University, Nagoya, Japan

**Keywords:** ascidian, sperm, egg, self-sterility, self-incompatibility, lysin, proteasome

## Abstract

Fertilization is one of the most important events in living organisms to generate a new life with a mixed genetic background. To achieve successful fertilization, sperm and eggs must undergo complex processes in a sequential order. Fertilization of marine invertebrate *Ciona intestinalis* type *A (Ciona robusta)* has been studied for more than a hundred years. Ascidian sperm are attracted by chemoattractants from eggs and bind to the vitelline coat. Subsequently, sperm penetrate through the vitelline coat proteolytically and finally fuse with the egg plasma membrane. Here, we summarize the fertilization mechanisms of ascidians, particularly from sperm-egg interactions to sperm penetration of the egg coat. Since ascidians are hermaphrodites, inbreeding depression is a serious problem. To avoid self-fertilization, ascidians possess a self-incompatibility system. In this review, we also describe the molecular mechanisms of the self-incompatibility system in *C. intestinalis* type A governed by three allelic gene pairs of *s-Themis* and *v-Themis*.

## Introduction

Fertilization of marine invertebrates has been extensively studied in classical research. This is not only because a large quantity of gametes can be obtained but also because *in vitro* fertilization experiments are easier in marine invertebrates than in mammals. Studying marine invertebrates led to the discovery of fundamental biological processes, including the fertilization process. The marine invertebrate *Ciona intestinalis* type A [another name: *Ciona robusta* (Brunetti et al., 2015)] is one of the model animals used to study many fields of biology, including reproductive biology (Sawada et al., 2001). The availability of draft genome sequences, transcriptomic and proteomics data and gene knockdown and gene editing techniques has supported the progress of *C. intestinalis* (type A) studies (Satou et al., 2001; Dehal et al., 2002; Shoguchi et al., 2006; Endo et al., 2011; Sasaki et al., 2014; Sasakura et al., 2017; Pickett and Zeller, 2018). Since ascidians are hermaphrodites, self-fertilization can occur. To avoid self-fertilization, many ascidians acquire a self-incompatibility (SI) system. After self/nonself-recognition, only nonself sperm can penetrate through the proteinaceous egg coat called the vitelline coat (VC) or chorion.

**FIGURE 1:**
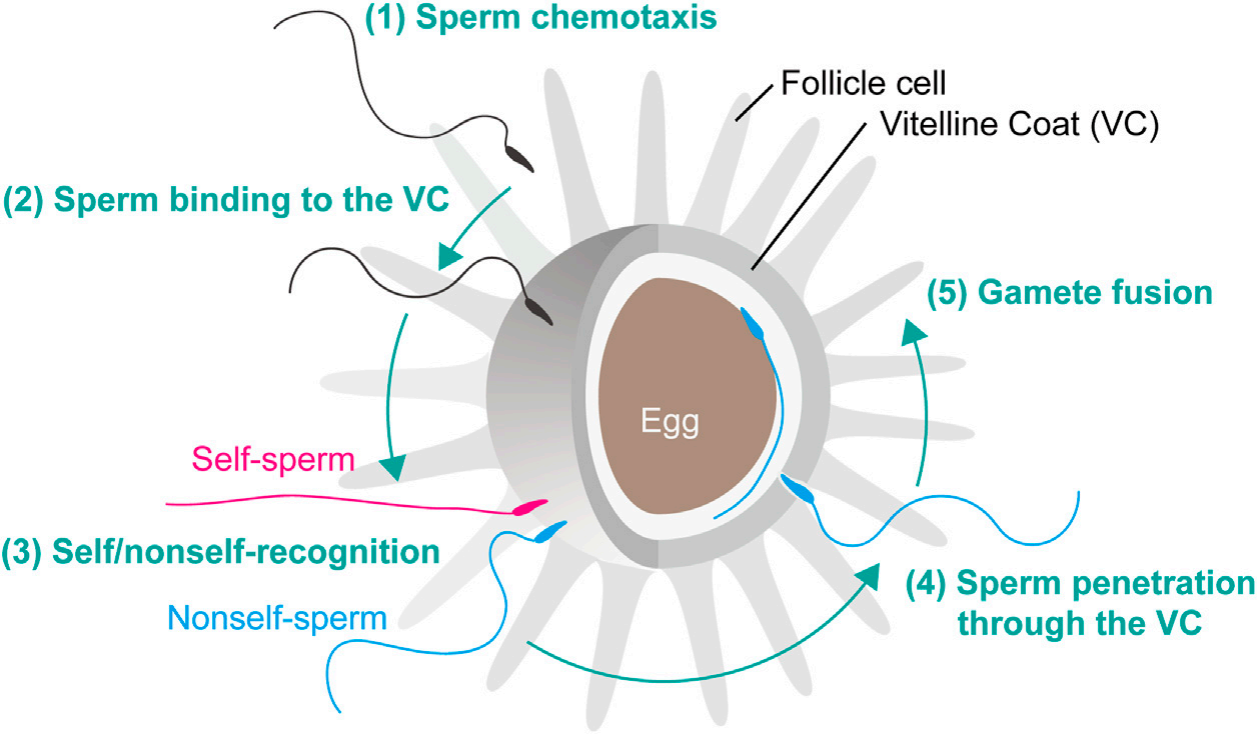
Fertilization steps of *C. intestinalis.* Fertilization is initiated by the following steps. (1) Sperm chemotaxis. (2) Binding of spermatozoa to the VC. (3) Interaction of self/nonself-recognition proteins that induce the signal transduction cascade leading to the SI response. (4) Penetration of nonself-recognized sperm through the VC. (5) Fusion of the egg and sperm plasma membranes resulting in fertilization and subsequent activation of zygote development.

Ascidian fertilization consists of five major steps: 1) sperm chemotaxis, 2) sperm binding to the VC, 3) self/nonself-recognition, 4) sperm penetration through the VC, 5) gamete fusion (Figure 1), similar to the fertilization processes as previously reviewed (Vacquier and Swanson, 2011; Gallo and Costantini, 2012). In *C. intestinalis* (type A), spermatozoa are attracted toward the eggs by sperm attractant SAAF, a sulfated steroid 3,4,7,26-tetrahydroxycholestane-3,26-disulfate (Yoshida et al., 2002). Transient intracellular Ca^2+^ increase is induced by SAAF gradient (Shiba et al., 2008), which is mediated by store-operated calcium channel (Yoshida et al., 2003) and sperm-membrane SAAF-binding protein, Ca-ATPase (Yoshida et al., 2018). After sperm chemotaxis, spermatozoa bind to the VC and undergo “sperm reaction”, which is characterized by vigorous movement on the VC and mitochondrial sliding and eventual shedding (Lambert and Epel, 1979; Lambert and Koch, 1988). Most animal spermatozoon besides teleosts has an acrosome at the tip of sperm head and undergoes acrosome reaction, an exocytosis of the acrosomal vesicle, upon sperm binding to the carbohydrate or proteinaceous egg coat. However, the acrosome of ascidian sperm is very tiny and it is still unclear whether the acrosome reaction takes place before (De Santis et al., 1980) or after (Fukumoto, 1988) sperm penetration of the VC. In this minireview, we describe our current understanding and future perspectives on ascidian fertilization, particularly in steps 2) to 4). After sperm penetration of the egg coat, gamete fusion takes place, which is mediated by sperm IZUMO1 and egg JUNO and CD9 in mice (see review: Okabe, 2018). However, the molecular basis of gamete fusion is poorly understood in ascidians. Recently, GCS1/Hap2 is reported to play a pivotal role in gamete fusion in plants and animals (see review: Mori et al., 2015), but the homologous genes and their functions are not known in ascidians or vertebrates.

## Gamete Interaction

### Sperm-Egg Interaction

After sperm attraction to the egg, gamete interaction takes place between the egg coat and sperm head (Figure 1). The egg coat is an extracellular matrix called the zona pellucida (ZP) in mammals and the vitelline envelope (VE) or VC in other animals. Structurally and evolutionally conserved egg coat proteins and ZP domain proteins are the major components of ZP, VE and VC (Aagaard et al., 2006; Goudet et al., 2008; Yamada et al., 2009). In mouse, the highly glycosylated proteins ZP1, ZP2 and ZP3, all of which contain a ZP domain at the C termini, are the building blocks of a filamentous network generated by polymerization of ZP domains (Bork and Sander, 1992; Jovine et al., 2002; Stsiapanava et al., 2020). Current studies have revealed that the N-terminal region of mouse and human ZP2 and the marine mollusk abalone VE receptor for lysin (VERL) crucially regulate gamete interactions (Gahlay et al., 2010; Avella et al., 2014; Raj et al., 2017). Similarly, the VC of ascidian eggs is constructed by ZP domain proteins (Sawada et al., 2002a; Kürn et al., 2007a; Yamada et al., 2009). In *C. intestinalis*, 11 ZP domain proteins have been identified by proteomic analysis of VC, and CiVC57 constitutes the most abundant ZP domain protein in VC. CiVC57 consists of a von Willebrand factor domain, 24 EGF-like repeats, a ZP domain, and a C-terminal transmembrane domain (Yamada et al., 2009). CiUrabin is an abundant protein on the surface of sperm, and in the lipid-raft-membrane fraction, it can bind to CiVC57, suggesting that CiVC57 and CiUrabin play a key role in gamete interaction (Yamaguchi et al., 2011; Nakazawa et al., 2015). CiUrabin belongs to a cysteine-rich secretory protein (CRISP) family containing a pathogenesis-related (PR) domain and a glycosylphosphatidylinositol (GPI)-anchor attachment site (Yamaguchi et al., 2011). Sperm CRISP family proteins participate in several steps in mammalian fertilization (Gonzalez et al., 2021). In particular, the PR domain of CRISP-1 is evolutionarily conserved and involved in sperm-egg binding (Ellerman et al., 2006). In addition, sperm-egg binding in another ascidian *Halocynthia roretzi* is mediated by the CRISP family protein HrUrabin (Urayama et al., 2008). Most likely, the interaction between CiVC57 and CiUrabin participates in the primary binding between sperm and VC in *C. intestinalis* (Yamaguchi et al., 2011; Saito et al., 2012).

It is also worth noting that *C. intestinalis* sperm α-L-fucosidase and L-fucosyl residues of glycoproteins on the VC play a key role in sperm binding to the VC for the following reasons (Hoshi et al., 1983; Hoshi et al., 1985). Sperm binding to the VC of glycerin-treated (glycerinated) eggs, whose VC sperm can bind to but not penetrate, was inhibited by L-fucose but not by D-fucose (Rosati and De Santis, 1980). In addition, α-L-fucosidase substrates (aryl α-L-fucoside) and competitive inhibitors (aryl β-L-fucoside) but not stereoisomer (aryl α-D-fucoside) blocked sperm binding to VC (Hoshi et al., 1983; Hoshi, 1985; Hoshi, 1986). α-L-fucosidase is located at the tip and the surface of the sperm head, as revealed by immunocytochemistry using several monoclonal antibodies raised against the purified enzyme. Whereas *C. intestinalis* sperm α-L-fucosidase showed an optimum pH for activity of approximately 3.9, it showed 2% or less of maximum activity in normal seawater (Hoshi et al., 1985). Sperm bound to VC in normal seawater detached within 7 min at 20°C but not at 0°C after decreasing the pH near the optimum pH of the enzyme (Hoshi et al., 1985; Hoshi, 1986). These results led to the conclusion that α-L-fucosidase at the tip of the sperm head is responsible for the primary binding of sperm to the fucosyl glycoprotein(s) on the VC in *C. intestinalis* (De Santis et al., 1983).

### Self/Nonself-Recognition Molecules

**FIGURE 2:**
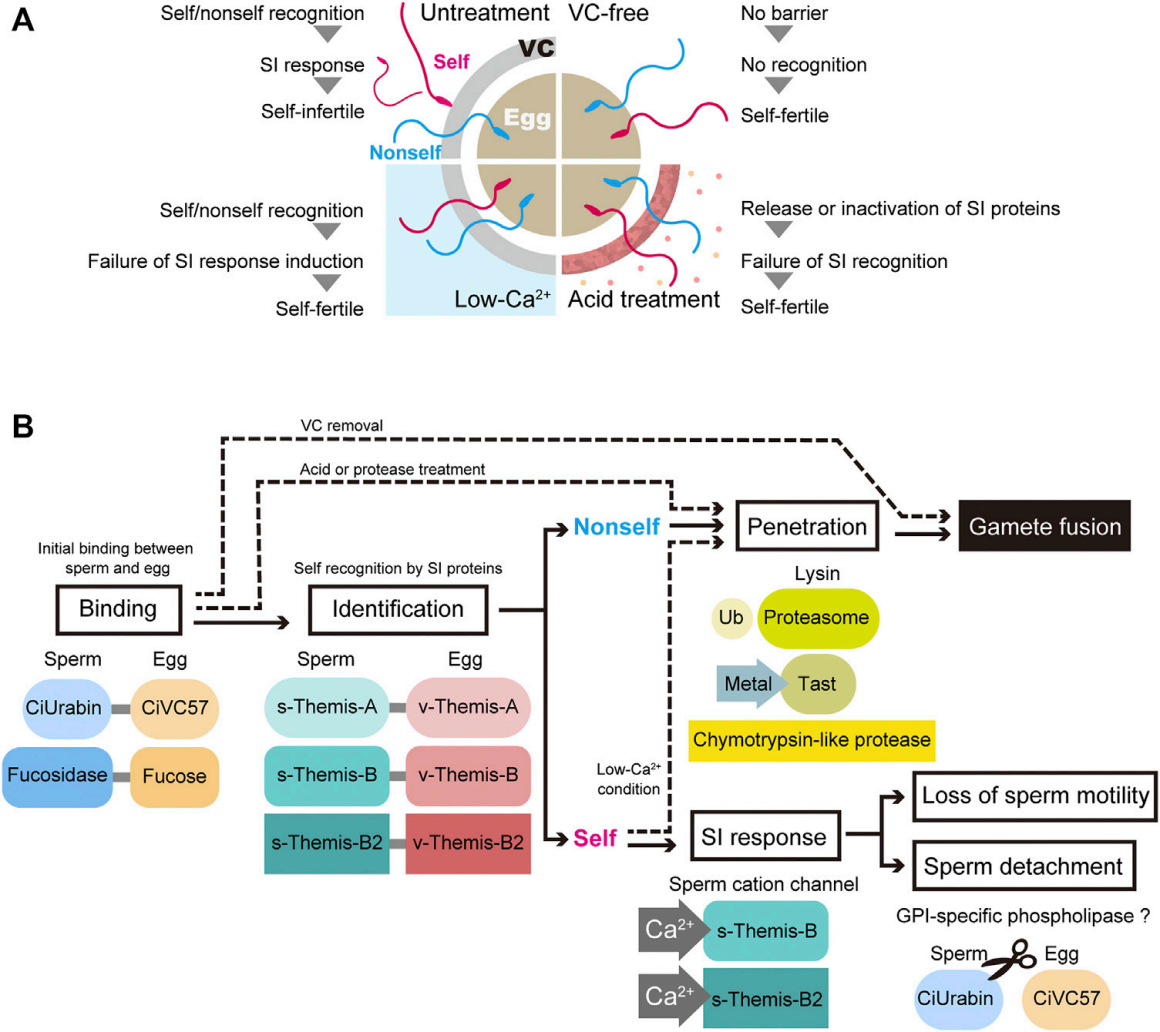
Current status of fertilization and self-incompatibility system. **(A)** Schematic representation of artificial self/nonself-fertilization in *C. intestinalis.* There are several ways to achieve artificial self-fertilization. Removing the VC and treating the VC with acidic seawater or proteases relieves the self-fertilization block. In addition, the inhibition of Ca^2+^ influx in spermatozoa, which is controlled by the external Ca^2+^ concentration, is sufficient to block the self-fertilization signal. **(B)** Gamete proteins involved in each step of fertilization are illustrated. Ub, ubiquitin; Tast, tunicate astacin-like metalloprotease with thrombospondin-type-1 repeat.

Shortly after sperm binding to the VC, a self/nonself-recognition process takes place on the VC (Morgan, 1939; Rosati and De Santis, 1978; Kawamura et al., 1987; Figure 1, Figure 2A). Identification of self/nonself-recognition molecules was attempted by several groups, and candidate molecules have been proposed (Kawamura et al., 1991; Pinto et al., 1995; Marino et al., 1998; Kürn et al., 2007a; Kürn et al., 2007b; Harada et al., 2008). Marino et al. proposed that autologous peptide-associating hsp70 on the surface of folliele cells participates in self/nonself-recognition during oogenesis, by analogy to antigenic peptide-presenting MHC (Marino et al., 1998). However, the peptides trapped by hsp70 have not been identified. Kawamura et al. found several factors in the acid-extract involved in allorecogniton, i.e., a non-allorecognizing glucose-enriched inhibitor of the gamete interaction and Glu/Gln-enriched peptide modulators, which function as cofactors in allorecognition of sperm receptors (Kawamura et al., 1991; Figure 2A). Khalturin and his colleague identified several genes expressed in oocytes and follicle cells that are highly polymorphic among individuals. They suggested that these highly polymorphic ZP domain proteins and sushi domain-containing protein vCRL1 may be the molecular basis for fertilization and allorecognition in *C. intestinalis* type B (Khalturin et al., 2005; Kürn et al., 2007a; Kürn et al., 2007b). However, their segregation and gene-knockdown analyses of *vCRL1* indicated that vCRL is important for the establishment and maintenance of the blood system, not in self/nonself-recognition (Sommer et al., 2012).

Self-sterility in *C. intestinalis* was first reported by Thomas Hunt Morgan, the founder of modern genetics, more than a hundred years ago (Morgan, 1910). Murabe and Hoshi (2002) and Harada et al. (2008) repeated Morgan’s experiments to determine whether crossing between sperm and eggs from selfed F1 siblings resulted in fertile or sterile offspring (Morgan, 1942; 1942), and they confirmed Morgan’s observation that one-way cross-sterility occurs between selfed F1 siblings, and this scarcely occurs under natural conditions (Murabe and Hoshi, 2002; Harada et al., 2008). Morgan concluded that the SI system is genetically controlled and hypothesized that this explains one-way cross sterility (Morgan, 1942). Morgan assumed that the “male” SI genes are expressed in haploids, while the “female” SI genes are expressed in diploids. This is called the “haploid sperm hypothesis”. Heterozygous individuals (A/a) release A-sperm and a-sperm, either of which can fertilize a homozygous individual’s eggs (a/a-eggs or A/A-eggs). On the other hand, homozygous individuals (A/A or a/a) release A-sperm or a-sperm alone, respectively, which cannot fertilize a heterozygous individual’s eggs (A/a-eggs), because both the A-sperm receptor and a-sperm receptor reside in the VC. Based on these criteria, Harada et al. carried out fertilization experiments between selfed F1 siblings and searched for a one-way cross-sterile combination. Seventy marker genes on 14 chromosomes were examined by PCR to determine whether they were homozygous or heterozygous (Harada et al., 2008). According to these strategies, two loci (locus A on chromosome 2q and locus B on chromosome 7q) were identified as loci responsible for SI. Among the approximately 20 genes in locus A, only one gene product (fibrinogen-like protein) was identified in the VC by proteome analysis (Yamada et al., 2009), and a polymorphic gene expressed in the testis was identified as a candidate sperm-side SI factor. These gene pairs were designated *v-Themis-A* and *s-Themis-A,* and two similar gene pairs were identified in locus B and named *s/v-Themis-B* and *s/v-Themis-B2* (Harada et al., 2008; Sawada et al., 2020). Taken together, there are three multiallelic pairs of SI candidate genes: egg-side genes (*v-Themis-A*, *v-Themis-B* and *v-Themis-B2*) and sperm-side genes (*s-Themis-A*, *s-Themis-B* and *s-Themis-B2*) (Harada et al., 2008; Yamada et al., 2009; Sawada et al., 2020). Interestingly, these are highly polymorphic genes and are tightly linked: *v-Themis* genes are encoded in the first intron of *s-Themis* genes in the opposite direction (Harada et al., 2008; Sawada et al., 2020). The sperm-side SI genes *s-Themis-A*, *B*, and *B2* show homology to mammalian *PKD1* or *PKDREJ*, which both contain a hypervariable region (HVR), receptor for egg jelly (REJ), G protein-coupled receptor proteolysis site (GPS), lipoxygenase homology 2 (LH2) domain, and 5 (in case of *s-Themis-A*) or 11 (in case of s*-Themis-B* and *s-Themis-B2*) pass transmembrane (TM) domain. Notably, *s-Themis-B* and *s-Themis-B2* possess a cation channel [polycystic kidney disease (PKD) channel] domain in their C-terminal regions (Harada et al., 2008; Sawada et al., 2020). When three allelic gene pairs were matched (in the case of the same haplotypes), even nonself-fertilization was strongly blocked (Sawada et al., 2020). In addition, by gene editing experiments, *s/v-Themis-A* genes and *s/v-Themis-B/B2* genes were found to be indispensable for self-sterility (Sawada et al., 2020). After sperm recognize the VC as self, drastic and acute Ca^2+^ influx occurs in spermatozoa, referred to as the SI response (Saito et al., 2012). This dramatic SI response causes sperm detachment from the VC and loss of sperm motility, because intracellular Ca^2+^ concentration regulates sperm behavior and flagellar beating. Although the SI response abolishes the fertility of self-recognized sperm, low-Ca^2+^ seawater enables self-fertilization (Hashimoto et al., submitted; Figures 2A,B). These results indicate that the increase in intracellular Ca^2+^ concentration triggers the SI response in spermatozoa.

Since the SI system is abolished by acid treatment of the VC, putative SI proteins might be released (Kawamura et al., 1991). Therefore, acid extracts of the VC were investigated by LC/MS, and Ci-v-Themis-like protein was identified as a new SI candidate protein (Otsuka et al., 2013). As the name indicates, Ci-v-Themis-like protein has a fibrinogen-like domain, similar to v-Themis-A, -B and -B2. However, Ci-v-Themis-like protein is not polymorphic among individuals. Although this protein is unlikely to participate in the SI system, it is probably involved in gamete binding or the assembly of v-Themis proteins on the VC (Otsuka et al., 2013).

Taken together, spermatozoa are equally attracted to self and nonself eggs and are capable of binding to the VC before recognizing the eggs as self or nonself. The interaction between sperm fucosidase and the VC fucose moiety must be maintained even after recognition as nonself, since nonself sperm binding to the VC is inhibited by fucose and fucose glycosides (Hoshi et al., 1983; Hoshi et al., 1985). Sperm fucosidase-fucose interactions may be involved in the interaction between s-Themis and v-Themis. On the other hand, the CiUrabin-CiVC57 interaction may be broken after sperm recognize the VC as self, since most self-sperm detach from the VC (Kawamura et al., 1987; Saito et al., 2012). In this process, GPI-specific phospholipase might be activated after sperm recognize the VC as self. Shortly after sperm binding to the VC, gametes undergo a judgment of self- or nonself-gametes using three allelic protein pairs of s-Themis and v-Themis, and only self-recognized sperm are rejected because self-recognition triggers the SI response (Figure 2B). s/v-Themis homologous genes were also identified in self-sterile species, such as *H. roretzi* and *C. savignyi*. However, participation of these proteins in SI is not known, since crossing experiments and genetic analysis have not been done. Since human sperm PKDREJ shows significant polymorphism (Hamm et al., 2007), it is an intriguing issue to clarify whether these variations are related to fertilization efficiency.

## Sperm Vitelline-Coat Lysins

Sperm utilize the lytic agent “lysin”, which enables sperm to penetrate through the proteinaceous egg coat (Figure 1). In ascidians, nonself-sperm lysin must be activated after self/nonself-recognition on the VC, which allows sperm to penetrate the VC. Generally, deuterostome spermatozoa appear to utilize enzymatic lysins, since protease inhibitors can inhibit fertilization, particularly sperm penetration of the ZP, VE or VC. In mammals, sperm acrosin, an acrosomal trypsin-like protease, is thought to be a zona-lysin (Hoshi, 1985). However, acrosin was found to be a nonessential gene for sperm penetration of the ZP and fertilization in mouse, as revealed by studies of *acrosin* knockout (KO) mice (Baba et al., 1994). In fact, acrosomal protease(s) other than acrosin are responsible for the sperm penetration process in mice (Yamagata et al., 1998). On the other hand, it was recently reported that *acrosin* is essential for hamster fertilization, more precisely sperm penetration of the ZP, by studying an *acrosin*-KO hamster model (Hirose et al., 2020). Therefore, whether acrosin is a zona-lysin depends on the species.

Hoshi et al. explored sperm proteases functioning as lysins using *Halocynthia roretzi*. Trypsin inhibitor (leupeptin) and chymotrypsin inhibitor (chymostatin) blocked the fertilization of intact eggs but not of VC-free eggs. Therefore, sperm trypsin-like and chymotrypsin-like proteases appear to participate in the sperm penetration of the VC, most likely as lysins (Hoshi et al., 1981). Sawada and his colleagues purified two trypsin-like proteases, ascidian acrosin and spermosin, from *H. roretzi* sperm extract (Sawada et al., 1984a). The involvement of both enzymes in fertilization was confirmed by comparing the abilities of various leupeptin analogs in inhibition of fertilization with their purified enzymatic activities (Sawada et al., 1984b). However, these enzymes showed no appreciable VC-degrading activity. Then, chymotrypsin-like protease was purified using Suc-Leu-Leu-Val-Tyr-MCA, the strongest inhibitor of fertilization among the chymotrypsin substrates tested, and the purified enzyme was identified as the proteasome (Sawada et al., 2002a). The purified proteasome from sperm can degrade the main component of the VC, HrVC70, after ubiquitination. Furthermore, anti-proteasome and anti-multiubiquitin antibodies and proteasome inhibitors inhibited fertilization. Lys-234 of HrVC70 appears to be ubiquitinated during fertilization, followed by degradation by the proteasome secreted upon sperm activation (sperm reaction) (Saitoh et al., 1993; Sawada et al., 2002a; Sakai et al., 2003). These results indicate that the sperm ubiquitin–proteasome system plays a pivotal role in fertilization, functioning as a VC lysin (Sawada et al., 2002b; Sawada, 2002). Notably, spermatozoa of mammals (Sutovsky et al., 2004), quail (Sasanami et al., 2012) and sea urchins (Yokota and Sawada, 2007) also utilize the sperm proteasome as egg-coat lysin.

In *C. intestinalis*, the effects of leupeptin and chymostatin on fertilization were examined (Hoshi, 1985). In this species, fertilization was inhibited by chymostatin but not by leupeptin, suggesting that *C. intestinalis* (Phlebobranch) and *H. roretzi* (Stolidobranch) utilize a different lysin system. A 24-kDa chymotrypsin-like protease was purified from *C. intestinalis* sperm, and the purified enzyme affected the outer layer of the VC, as revealed by electron microscopy (Marino et al., 1992). Analogous to *H. roretzi*, the participation of the sperm proteasome in fertilization was investigated in *C. intestinalis*. The proteasome inhibitors MG115 and MG132 inhibited the fertilization of intact eggs but not VC-free eggs (Sawada et al., 1998). In addition, these inhibitors showed no appreciable inhibition of sperm binding to the VC of glycerinated eggs. These results suggest that the proteasome plays a key role in sperm penetration through the VC but not in sperm binding to the VC (Figure 2B). Further studies are needed to clarify the target VC protein(s) of these proteases.

To investigate whether these proteases are exposed to the plasma membrane of the sperm head, proteomic analysis of sperm surface proteins from *C. intestinalis* was performed (Nakazawa et al., 2015). Unexpectedly, chymotrypsin-like protease or proteasome subunits were not identified under the conditions tested. Instead, several metalloproteases, which are referred to as “Tast (tunicate astacin-like metalloprotease with thrombospondin type 1 repeat)”, were identified as the major proteases (Nakazawa et al., 2019). The involvement of metalloproteases in fertilization was tested by the metalloprotease inhibitor GM6001. GM6001 strongly inhibited the fertilization of intact eggs but not VC-free eggs, and GM6001 did not inhibit sperm binding to the VC of glycerinated eggs. Furthermore, when isolated VC was incubated with intact sperm, several VC proteins, including CiVC57, were degraded and inhibited by GM6001. Therefore, Tasts are promising candidates for VC lysin, although we cannot exclude the possibility that the 24-kDa chymotrypsin-like protease and the proteasome are also involved in sperm penetration of the VC. Generally, metalloproteases, such as matrix metalloproteases, or the proteasome are very powerful tools to degrade insoluble proteins. It is interesting to note that several egg coat-targeting proteases, such as mouse egg ovastacin (Burkart et al., 2012) and medaka hatching enzymes (Yasumasu et al., 1992), belong to astacin-like metalloproteases. Further detailed studies on the molecular mechanisms of degradation of VC proteins by sperm proteases functioning as lysins remain to be elucidated.

## Concluding Remarks

We summarized the current working hypothesis of the mechanisms of fertilization in *C. intestinalis,* particularly from the sperm binding to the VC to the sperm penetration through the VC (Figure 2B). Shortly after sperm binding to the VC, which is mediated by interactions between CiUrabin and CiVC57, self/nonself-recognition molecules may be recruited to the binding site. When three allelic pairs (haplotypes), i.e., s/v-Themis-A, s/v-Themis-B, and s/v-Themis-B2, are matched (self-recognition), drastic Ca^2+^ influx occurs in spermatozoa, and the self-recognized sperm detaches from the VC or quits moving on the VC. Recognizing autologous proteins rather than an enormous number of allogeneic proteins is much easier and more reasonable. Genome editing experiments clearly demonstrated that s/v-Themis-A and s/v-Themis-B/B2 are indispensable for self/nonself-recognition. In addition, s-Themis-B and -B2 were more crucial than s-Themis-A in the SI system (Sawada et al., 2020). This is not at variance with the fact that both s-Themis-B and s-Themis-B2 contain a cation channel at their C-termini that is probably involved in Ca^2+^ influx. Similar to the SI system in Papaveraceae, Ca^2+^ influx in spermatozoa may induce sperm cell apoptosis. Further studies are necessary to demonstrate the protein–protein interaction between s-Themis and v-Themis. Intracellular signal transduction after the SI response is also an intriguing issue to be investigated. In the lysin system, we revealed the importance of a novel astacin-like metalloprotease (Tast) in sperm passage of the VC. Elucidating the physiological substrates of sperm Tasts and proteasomes are also important issues that remain to be clarified.
